# Detection of Lineage IV Peste Des Petits Ruminants Virus by RT-qPCR Assay via Targeting the Hemagglutinin Gene

**DOI:** 10.3390/v17070976

**Published:** 2025-07-12

**Authors:** Jiao Xu, Qinghua Wang, Jiarong Yu, Yingli Wang, Huicong Li, Lin Li, Jingyue Bao, Zhiliang Wang

**Affiliations:** 1 China Animal Health and Epidemiology Center, Qingdao, 266000, China; xujiao@cahec.cn (J.X.);; 2 College of Veterinary Medicine, Qingdao Agricultural University, Qingdao, 266109, China

**Keywords:** peste des petits ruminants virus, reverse transcription quantitative polymerase chain reaction, detection, diagnosis

## Abstract

Peste des petits ruminants virus (PPRV) has been classified into four lineages based on the nucleocapsid and fusion genes, with lineage IV strains being the most widely distributed. In Africa, recent epidemiological data revealed that PPRV lineage IV is increasingly displacing other lineages in prevalence, suggesting a competitive advantage in viral transmission and adaptability. Moreover, a lineage IV strain was the only confirmed strain in Europe and Asia. In this study, a one-step Taqman quantitative real-time reverse transcription polymerase chain reaction (RT-qPCR) assay for lineage IV PPRV was established by targeting the hemagglutinin (H) gene. The results indicated that this method could detect approximately six copies of PPRV RNA, indicating high sensitivity. No cross-reactions with related viruses or other lineages of PPRV were observed. The results of a repeatability test indicated that the coefficient of variation values were low in both the inter-assay and intra-assay experimental groups. Detection of field samples indicated that all positive samples could be detected successfully using the developed method. This RT-qPCR assay provides a valuable tool to facilitate targeted surveillance and rapid differential diagnosis in regions with active circulation of PPRV lineage IV, enabling timely epidemiological investigations and strain-specific identification.

## 1. Introduction

Peste des petits ruminants (PPR) is a highly contagious disease caused by the peste des petits ruminants virus (PPRV), which primarily affects goats, sheep, and small wild ruminants. The incubation period of PPR is typically 4 to 6 days but may range from 3 to 10 days. Fever; increased ocular, oral, and nasal discharges; gastritis; diarrhea; and pneumonia are usually observed in infected animals [1]. PPRV belongs to the species Small ruminant morbillivirus, genus Morbilliviruses, subfamily Orthoparamyxovirinae, and family Paramyxoviridae and has a linear negative-stranded RNA genome [2,3]. The PPRV genome encodes six structural proteins, including the nucleocapsid (N), matrix (M), phosphoprotein (P), fusion (F), hemagglutinin (H), polymerase (L) proteins, and two nonstructural proteins (C and V), in the order of 3′N-P/C/V-M-F-H-L-5′ [4]. Although only one serotype of PPRV was confirmed, according to the N or F gene, PPRV can be classified into four lineages [2]. Lineages I and II are mainly distributed in West Africa, lineage III is mainly distributed in East Africa, and lineage IV is widely distributed in the Arabian Peninsula, the Middle East, and Southern and Eastern Asia [5]. Huge economic losses have been associated with PPRV, with direct or indirect losses reaching USD 2.1 billion each year. Once newly introduced, PPRV can infect up to 90% of a herd and cause 70% death of infected animals. Under the Global Framework for the Progressive Control of Transboundary Animal Disease (GF-TADs), the World Organization for Animal Health (WOAH) has identified PPR for global control and eradication by 2030.

PPR was first discovered in Cote d’Ivoire in 1942 and mainly circulated in Africa and Asia. However, it continues to spread to new areas despite various control methods having been implemented. In July 2024, PPR re-emerged in Europe (Bulgaria previously reported one case of PPR in 2018), including cases in Greece, Romania, and Hungary. Consequently, PPR epidemic-free certification of these countries was suspended, and according to the World Animal Health Information System (WAHIS), PPR is still circulating in these countries, indicating that the area influenced by PPRV has further expanded. The results of viral phylogenetic analysis indicated that the virus isolated from Greece and Romania belonged to lineage IV and shared high genetic similarity with the strains circulating in Georgia [6]. In 2007, China first reported PPR in Tibet, and in 2013, PPR re-emerged in the Xinjiang Uygur Autonomous Region and rapidly spread to almost all provinces [7,8]. Subsequently, various control methods were implemented, including culling, restriction on transportation of susceptible animals, compulsory vaccination, and active surveillance. Benefitting from these measures, the PPR situation in China has stabilized in recent years, with only a few PPR cases being reported.

It is very difficult to differentiate PPR from rinderpest because of their similar clinical sign presentations; therefore, all PPR cases should be confirmed via laboratory diagnostic methods. Timely diagnosis is critical to curbing the transmission of PPRV. Viral isolation is regarded as the gold standard among all diagnostic methods; however, it is time-consuming and is technically difficult, which has limited its wide application [9]. Enzyme-linked immunosorbent assays (ELISAs) are mainly used for PPRV antibody detection. However, using current technology, it is impossible to distinguish between antibodies produced by natural wild virus infection and those generated through vaccination. Consequently, ELISA is usually applied to monitor antibody levels and for immune efficacy evaluation. Several new detection methods for PPR were also established recently, such as loop-mediated isothermal amplification (LAMP) [10] and clustered regularly interspaced short palindromic repeats (CRISPR) [11], which are easy to operate and observe; however, their cost remains relatively high compared with conventional methods. Quantitative real-time reverse transcription polymerase chain reaction (RT-qPCR) is still the most commonly used method for PPRV detection and is recommended by WOAH because of its accuracy, sensitivity, and economy. Compared to conventional PCR, it exhibits higher sensitivity, simpler operation, and requires less time. The PPRV N gene is preferentially targeted in RT-qPCR assays because of its proximity to the viral promoter region, which is responsible for the N protein’s status as the most abundantly expressed viral protein during replication [3,12]. The F gene has also been selected as a target because of its conserved sequence. Several primers and probes with high sensitivity and specificity are available to detect PPRV. A rapid RT-qPCR assay based on the N and F genes, established by Batten and Flannery, could detect 10 viral genomic copies of PPRV, and all four lineages could be successfully detected [13]. Kwiatek et al. established an RT-qPCR method based on the N gene, with a detection limit of approximately 32 viral genome copies for all lineages of PPRV [14]. Based on the N gene, Polci designed primers and a probe that could detect approximately 20 copies of the virus with a 95% probability, and no amplification signals were recorded when the method was applied to viruses closely related or clinically similar to PPRV- or PPR-negative blood samples [15].

In Africa, lineages I to III viruses were widespread before 2008. However, lineage IV was found to replace the other lineages and spread widely in relevant African countries since then [16,17,18,19]. In the Arabian Peninsula, the only PPRV that circulated before was lineage III. However, no cases of lineage III were reported in recent years; conversely, the PPRV cases reported in the vast majority of Asian countries belong to lineage IV [20]. In Europe, the circulating PPRV in a newly affected region was also confirmed as lineage IV [6]. Lineage IV of PPRV has emerged as the predominant strain with the broadest global distribution. Several years ago, we designed a pair of primers and a probe for PPRV detection based on the N gene [21]. However, according to in silico evaluation of PPRV-RT-qPCR assays performed by Flannery [13], the sensitivity of this primer pair was relatively low when compared with other assays. Consequently, in this study, we aimed to develop a novel H gene-based RT-qPCR assay for PPRV lineage IV and systematically evaluated its diagnostic performance through sensitivity analysis, specificity assessment, and repeatability testing. The established method demonstrated significant potential as an effective diagnostic alternative for PPR surveillance and control programs.

## 2. Materials and Methods

### 2.1. Primer and Probe Design

PPRV full genomic sequences of lineages I to IV were collected from GenBank and aligned using Snapgene software (GSL Biotech LLC, Boston, MA, USA). The primers and probe were designed based on the conserved regions of the PPRV H gene from lineage IV PPRV. The forward primer matched position 7974–7996 (5′-AACGTGTCCTCAGTGTTTACCRT-3′); the Taqman probe bound to position 8014–8043 (FAM-CGGAAGAACATATACYGTCTGGAGATCCG-BHQ1), whereas the reverse primer matched position 8076–8101 (5′-ATCTCGAAGACTCTTAAAAATGGCC-3′) (Reference strain: China/XJYL/2013, GenBank: KM091959.1).

### 2.2. Virus and Plasmids

Goat pox virus (GPV), Orf virus (ORFV) and Foot-and-Mouth disease virus (FMDV) were collected from clinical samples. PPRV China/XJYL/2013 (lineage IV) and China/Tibet/2007 (lineage IV) were stored and prepared at the National Reference Laboratory for Peste des Petits Ruminants (at the China Animal Health and Epidemiology Center (CAHEC), Qingdao, China). Partial H genes from PPRV/Cote_dIvoire/1989 (lineage I), Ghana/NK1/2010 (lineage II), SnDK11/13 (lineage II), KN5/2011 (lineage III), UAE 1986 (lineage III), PPRV/Oman 1983 (lineage III), and the Mprocco 2008 strain (lineage IV) were commercially synthesized and cloned into vector pUC57. All plasmids were constructed and verified by Shanghai Sangon Biotechnology Company (Shanghai, China). The PPRV/XJYL/2013 virus standard used for sensitivity and repeatability assays was provided from the National Reference Laboratory for Peste des Petits Ruminants.

### 2.3. RNA Extraction and Real-Time Quantitative RT-PCR

All clinical samples were processed in a biosafety level III (BSL-3) laboratory. Viral RNA was extracted using a magnetic bead-based viral DNA/RNA extraction kit (Xi’an Tianlong Science and Technology Co., Ltd., Xi’an, China) according to the manufacturer’s instructions. qPCR amplification and detection were performed using a Bio-Rad PCR machine using a one-step RT-qPCR kit (RR600A, Takara, Shiga, Japan). Each reaction system comprised 25 μL: 12.5 μL of one-step PrimeScript III RT-qPCR Mix (1×), 1 μL of forward primer (0.4 μM), 1 μL of reverse primer (0.4 μM), 0.5 μL of probe (0.2 μM), 0.5 μL of ROX reference dye (50 nM), 3 μL of the RNA sample, and 6.5 μL of RNA-free water. All samples were detected in duplicate. The cycling conditions were set as follows: reverse transcription at 50 °C for 10 min, followed by reverse transcriptase inactivation and DNA polymerase activation at 95 °C for 5 min, and 40 cycles of amplification (denaturation at 95 °C for 15 s and annealing at 60 °C for 30 s). Fluorescence signals were collected at 60 °C in each cycle, and cycle thresholds (Cts) were assigned to each sample in the exponential phase of the amplification plot of each cycle.

### 2.4. Sensitivity Test

Viral standard nucleic acids were serially diluted 10-fold to concentrations ranging from 6 × 10^0^ to 10^6^ copies/μL, respectively. The diluted viral nucleic acids were used as templates for detection using the designed primers and probe.

### 2.5. Repeatability Test

Viral standard nucleic acids were serially diluted 10-fold to concentrations ranging from 6 × 10^0^ to 10^6^ copies/μL, respectively. Different concentrations of viral nucleic acid were detected using primer and probe, and the inter-assay repeatability and intra-assay repeatability were all evaluated, respectively.

### 2.6. Specificity Test

The specificity of the assay was assessed by detecting various viruses, including GPV, ORFV, FMDV, PPRV China/XJYL/2013 and China/Tibet/2007 strains, PPRV/Cote_dIvoire/1989, Ghana/NK1/2010, SnDK11/13, KN5/2011, UAE 1986, PPRV/Oman 1983, and Mprocco 2008 plasmids.

### 2.7. Field Samples

One hundred field samples comprising swabs and tissues were collected by our laboratory. PPRV was inactivated using 1% (*w/v*) NaOH in a BSL-3 laboratory, and viral RNA was extracted, followed by reverse transcription in a BSL-2 laboratory. All clinical samples were detected and confirmed as PPRV-positive or PPRV-negative using conventional RT-PCR [22] before the primers and probe designed in this study were used for the detection of these samples.

## 3. Results

### 3.1. Multiple Sequence Alignments of Primers and Probe Target Sites

**Figure 1:**
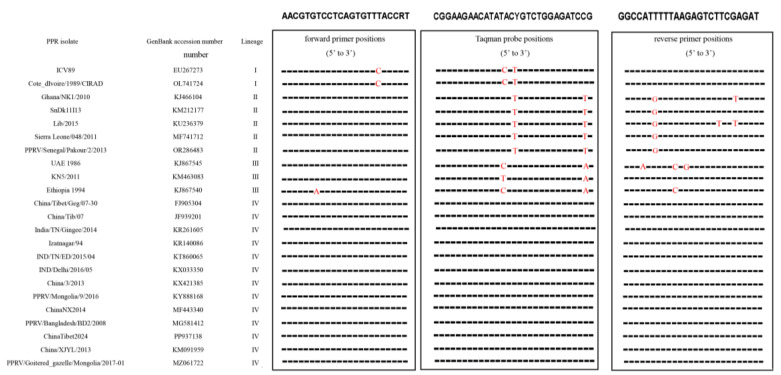
Alignment of the primer and probe targeted to the gene among multiple PPRV strains. The sequences of the forward primers, probe and reverse primer are shown in boxes; red represented different nucleotides from the primer and probe.

The sequence alignment analysis presented in Figure 1 demonstrates that both the primers and the probe developed in this study exhibit high specificity for conserved genomic regions within PPRV lineage IV.

### 3.2. Sensitivity and Standard Curve of the Real-Time RT-qPCR

**Figure 2:**
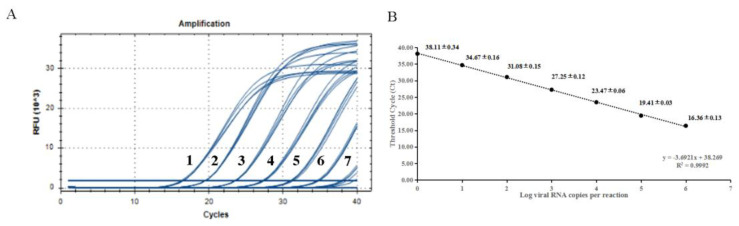
Sensitivity tests and standard curve for RT-qPCR based on the H gene. (**A**) Amplification curves of templates with different concentrations, (**B**) Standard curve of developed method. The data for each concentration is presented as CT value ± SD. The lowest copy number that could be determined was up to 6 copies/μL; the optical standard formula is y = −3.6921x + 38.269, and the correlation coefficient is 0.9992.

The 10-fold gradient dilutions of the China/XJYL/2013 strain-derived RNA were detected. The minimum template concentration detected by this method was 6 copies of RNA, with a corresponding Ct value of 38.11. The dynamic range of the assay over a 10-log-unit span of viral RNA concentrations ranged from 6 to 6 × 10^6^ RNA copies/μL. The standard curve for the H-gene RT-qPCR assay was also performed. (Figure 2). The cut-off value was determined to be 38.69 through testing of negative samples.

### 3.3. Repeatability of the Real-Time RT-qPCR

Different copies of PPRV China/XJYL/2013 RNA were assayed for the repeatability test. The results showed that for the intra-assay repeatability, the CV values were all less than 1.50%. The CV values of the inter-assay repeatability were all less than 1.67% (Table 1).

### 3.4. Specificity of the Real-Time RT-qPCR

Results indicated that only PPRV China/XJYL/2013, China/Tibet/2007, and Morocco 2008 were detected by a developed method, and no cross-reaction with other viruses or plasmids was observed (Table 2).

### 3.5. Fields Samples

One hundred field samples collected from a national epidemiological investigation project on PPR or reported PPR cases in China were detected using conventional RT-PCR and the novel RT-qPCR method, respectively. The results indicated that all the positive samples confirmed by conventional RT-PCR were detected successfully using the novel method, demonstrating 100% correlation (Table 3).

## 4. Discussion

PPR is classified as a list A disease in China because of its biological risk and threat to domestic and wild small ruminants. Since the outbreak of PPR in 2007, a national epidemiological investigation project on PPR has been conducted in all provinces, autonomous regions, and municipalities. Compulsory vaccination has also been implemented in almost all areas in China since then. The PPR attenuated vaccine developed using the Nigeria 75/1 strain has been widely applied in China and has been confirmed to confer strong protective immunity with a long duration in sheep and goats [23]. The PPR Monitoring and Assessment Tool (PMAT) is based on four different stages identified in the Global Strategy for the progressive control and eradication of PPR, which correspond to a combination of decreasing levels of epidemiological risk and increasing levels of prevention and control. Vaccination is one of the key tools to control PPR and was identified as the main option in stage 2 and stage 3 of PMAT. However, in stage 4 (post-eradication), vaccinations need to be suspended and the capacity of laboratory diagnosis needs to be strengthened. In addition, various wild ruminants demonstrated susceptibility to PPR, including gemsbok, goitered gazelle, bharal, alpine ibex, and argali [9,24,25,26]. In contrast to domestic animals, wild populations present unique challenges for PPR vaccination because of their limited human contact and free-ranging behaviors. This barrier creates a critical gap in current epidemic prevention and control strategies, particularly with regard to effective immunization coverage. To address this vulnerability, implementation of active surveillance via advanced diagnostic technologies with enhanced sensitivity and specificity becomes imperative, enabling accurate detection and surveillance of PPR virus circulation in wild reservoirs.

As previously mentioned, the N and F genes have been commonly selected as potential targets to develop PPRV detection methods. Mahapatra developed a nested PCR method based on the N gene, which demonstrated high sensitivity in detecting all PPRV lineages [27]. Zhang established a kind of real-time reverse transcription recombinase-aided amplification (RT-RAA) assay based on the N gene, which could detect 103 copies of PPRV, with 99.4% concordance with conventional RT-PCR [28]. Additionally, Ke designed a lyophilized real-time fluorescent PCR assay targeting the F gene, which simultaneously detected 11 pathogens affecting sheep and goats [29]. During PPRV infection, most of the neutralizing antibodies are directed against the surface hemagglutinin (H) glycoprotein H [30]. The H glycoprotein serves as a principal target for neutralizing antibodies, making it a preferred candidate to develop serological detection methods, such as blocking and competitive ELISA assays [31,32]. However, despite the protein’s immunodominance in humoral responses, the H gene has been underutilized in nucleic acid-based diagnostic platforms compared with the more frequently targeted N and F genes because of its low sequence conservation. An earlier RT-qPCR assay targeting the H gene was evaluated; however, computational analysis using PCRv software revealed its lower sensitivity compared with that of N or F gene-based detection methods [13]. To provide a new choice for PPRV diagnosis, herein, we developed a novel H gene-specific RT-qPCR assay, which demonstrated significantly improved sensitivity and specificity. This advance establishes a reliable molecular detection tool that complements existing PPR diagnostic approaches while expanding the genetic targets available for comprehensive outbreak monitoring.

Lineage IV of PPRV has become the main epidemic strain around the world in recent years and thus should be paid more attention. According to related studies, no lineage I strains have been identified in the last 10 years, whereas lineage II and III strains were occasionally detected in some African countries [33,34,35,36]. As a transboundary animal disease, PPRV may be introduced into previously unaffected regions through the movement and commercial trade of live animals. Consequently, a rapid diagnosis method applicable for PPRV lineage IV is essential for the control of PPR. RT-qPCR has been widely used for PPR diagnosis and will continue to contribute to the global eradication program. RT-qPCR methods are usually evaluated according to their specificity and sensitivity. According to our results, the new method could detect as few as six copies of PPRV, showing higher sensitivity compared with other PPRV detection assays. PPR is sometimes misdiagnosed as other diseases because of their similar symptoms. Importantly, the developed method enables accurate differentiation of PPR from other prevalent pathologies affecting small ruminants. The low intra-assay and inter-assay CV values observed in the repeatability tests demonstrate the stability of our method, which is essential for a robust and practical detection method. However, some limitations of our research should be addressed here. In our research, plasmids served as a practical alternative for accessing genetic material from economically significant viruses. While this approach allowed us to bypass the reverse transcription (RT) step—effectively converting the RT-qPCR into a standard qPCR assay—one potential solution would be to generate RNA templates via in vitro transcription of the plasmids. However, this method could complicate the evaluation of certain RT-qPCR performance characteristics. Additionally, as noted in the manuscript, another limitation is that we did not benchmark our assay against the virus isolation, which is regarded as the gold standard for PPRV detection.

In conclusion, we established a novel RT-qPCR method with high sensitivity and specificity, which could detect PPRV lineage IV. The assay enables virus detection at the early stage of infection or in the presence of low virus levels, thus providing a powerful diagnostic instrument to aid the prevention and control of this animal disease.

## Figures and Tables

**Table 1:** The inter-assay (upper) and intra-assay (lower) repeatability of developed method.

Number	Viral RNA Copies	Ct				Mean Ct	SD	CV
1	6 × 10^6^	17.89	17.37	17.54	17.28	17.52	0.27	1.54%
2	6 × 10^5^	20.34	20.3	20.36	20.23	20.31	0.06	0.28%
3	6 × 10^4^	24.46	24.42	24.3	24.37	24.39	0.07	0.28%
4	6 × 10^3^	28.01	28.26	28.02	28.12	28.10	0.12	0.41%
5	6 × 10^2^	31.97	31.8	31.74	31.64	31.79	0.14	0.44%
6	6 × 10^1^	36.06	34.71	35.83	35.4	35.50	0.59	1.67%
7	6 × 10^0^	39.94	38.82	38.88	38.92	39.14	0.46	1.18%
Number	Viral RNA copies	Ct				Mean Ct	SD	CV
1	6 × 10^6^	18.65	18.63	18.32	18.54	18.54	0.15	0.82%
2	6 × 10^5^	22.47	22.59	22.49	22.47	22.51	0.06	0.26%
3	6 × 10^4^	26.54	26.64	26.71	26.57	26.62	0.08	0.29%
4	6 × 10^3^	29.99	30.1	29.83	30.06	30.00	0.12	0.40%
5	6 × 10^2^	33.47	33.89	33.45	33.52	33.58	0.21	0.62%
6	6 × 10^1^	37.76	36.59	37.52	36.81	37.17	0.56	1.50%
7	6 × 10^0^	39.64	39.63	39.78	39.5	39.64	0.14	0.35%

**Table 2:** The specificity of developed method.

Number	Name of Strains	GenBank Accession Number	Lineage	Results	Ct	Template
1	China/Tibet/2007	FJ905304	IV	+	18.99	Viral cDNA
2	China/XJYL/2013	KM091959	IV	+	22.12	Viral cDNA
3	Morocco 2008	KC594074	IV	+	25.44	Viral cDNA
4	PPRV/Oman 1983	KJ867544	III	-	Undetected	Plasmid
5	UAE 1986	KJ867545	III	-	Undetected	Plasmid
6	KN5/2011	KM463083	III	-	Undetected	Plasmid
7	Ghana/NK1/2010	KJ466104	II	-	Undetected	Plasmid
8	SnDK11/13	KM212177	II	-	Undetected	Plasmid
9	PPRV/Cote_d Ivoire/1989	EU267273	I	-	Undetected	Plasmid
10	GPV	/	/	-	Undetected	Plasmid
11	ORFV	/	/	-	Undetected	Plasmid
12	FMDV	/	/	-	Undetected	Plasmid

**Table 3:** Detective results of fields samples.

Sample ID			Type of Samples			Collection Date and Place			Conventional RT-PCR			RT-qPCR (Ct Value)		
1	2	3	swab	lymph node	swab	2007.8 Tibet Autonomous Region	2007.9 Tibet Autonomous Region	2007.9 Tibet Autonomous Region	−	−	−	−	−	−
4	5	6	swab	swab	swab	2007.9 Tibet Autonomous Region	2008.1 Tibet Autonomous Region	2010.2 Tibet Autonomous Region	+	+	+	28.15	16.55	22.65
7	8	9	lymph node	lymph node	swab	2010.3 Tibet Autonomous Region	2013.2 Xinjiang Uygur Autonomous Region	2013.2 Xinjiang Uygur Autonomous Region	−	+	+	−	28.95	27.22
10	11	12	spleen	spleen	spleen	2014.1 Gansu	2014.1 Gansu	2014.2 Inner Mongolia Autonomous Region	+	−	−	30.12	−	−
13	14	15	lymph node	swab	swab	2014.2 Ningxia	2014.2 Ningxia	2014.3 Liaoning	−	+	−	−	28.17	−
16	17	18	swab	swab	lymph node	2014.3 Liaoning	2014.3 Hunan	2014.3 Hunan	+	−	−	26.48	−	−
19	20	21	lymph node	lymph node	lymph node	2014.3 Anhui	2014.3 Anhui	2014.3 Jiangsu	+	+	−	31.22	24.96	−
22	23	24	lymph node	lymph node	lymph node	2014.3 Jiangsu	2014.3 Jiangxi	2014.3 Jiangxi	−	−	−	−	−	−
25	26	27	swab	rumen	lymph node	2014.3 Guangxi	2014.3 Guangxi	2014.3 Guangxi	+	−	−	30.05	−	−
28	29	30	swab	swab	swab	2014.3 Heilongjiang	2014.3 Heilongjiang	2014.3 Heilongjiang	−	+	−	−	18.64	−
31	32	33	swab	swab	swab	2014.3 Jilin	2014.3 Jilin	2014.4 Shanxi	−	+	−	−	19.45	−
34	35	36	swab	lymph node	lymph node	2014.4 Shanxi	2014.4 Chongqing	2014.4 Chongqing	+	−	+	14.65	−	23.21
37	38	39	lymph node	bronchus	lymph node	2014.4 Chongqing	2014.4 Chongqing	2014.4 Chongqing	−	−	+	−	−	24.02
40	41	42	bronchus	lymph node	lymph node	2014.4 Zhejiang	2014.4 Zhejiang	2014.4 Zhejiang	−	+	+	−	31.25	32.05
43	44	45	bronchus	lymph node	lymph node	2014.4 Zhejiang	2014.4 Chongqing	2014.4 Chongqing	−	+	−	−	27.52	−
46	47	48	swab	lung	lung	2014.4 Sichuan	2014.4 Sichuan	2014.4 Sichuan	+	−	−	33.01	−	−
49	50	51	swab	swab	swab	2014.4 Sichuan	2014.4 Sichuan	2014.4 Sichuan	−	+	+	−	27.28	22.13
52	53	54	swab	swab	swab	2014.4 Zhejiang	2014.4 Zhejiang	2014.4 Hubei	+	+	+	30.32	21.57	23.85
55	56	57	swab	lymph node	lymph node	2014.4 Hubei	2014.4 Hubei	2014.4 Anhui	−	−	−	−	−	−
58	59	60	lymph node	lymph node	lymph node	2014.4 Anhui	2014.4 Anhui	2014.4 Heilongjiang	−	+	−	−	24.92	−
61	62	63	lymph node	lymph node	lymph node	2014.4 Heilongjiang	2014.4 Heilongjiang	2014.4 Jiangxi	−	−	−	−	−	−
64	65	66	lymph node	lymph node	lymph node	2014.5 Yunnan	2014.5 Yunnan	2014.6 Shaanxi	+	−	+	31.72	−	26.26
67	68	69	lymph node	lymph node	lymph node	2014.6 Shaanxi	2014.6 Shaanxi	2014.6 Shaanxi	−	−	+	−	−	25.41
70	71	72	swab	lymph node	lymph node	2014.8 Guizhou	2014.8 Guizhou	2014.8 Guizhou	−	+	−	−	19.68	−
73	74	75	swab	swab	swab	2015.4 Guizhou	2015.4 Guizhou	2015.8 Ningxia	+	+	−	17.99	25.62	−
76	77	78	lymph node	lymph node	lymph node	2015.8 Ningxia	2016.11 Ningxia	2016.11 Ningxia	+	+	+	24.72	26.38	29.85
79	80	81	lymph node	trachea	lymph node	2017.3 Hunan	2017.3 Hunan	2017.3 Hunan	−	+	−	−	31.85	−
82	83	84	swab	lymph node	lymph node	2018.2 Qinghai	2018.2 Qinghai	2018.2 Qinghai	−	−	+	−	−	30.84
85	86	87	swab	swab	swab	2018.5 Jiangsu	2019.1 Ningxia	2019.1 Ningxia	−	−	−	−	−	−
88	89	90	swab	trachea	swab	2020.5 Liaoning	2020.5 Liaoning	2020.5 Liaoning	−	+	−	−	19.88	−
91	92	93	swab	swab	swab	2021.2 Xinjiang Uygur Autonomous Region	2021.2 Xinjiang Uygur Autonomous Region	2021.2 Xinjiang Uygur Autonomous Region	+	+	+	16.38	24.52	26.84
94	95	96	lymph node	lymph node	lymph node	2021.3 Tibet Autonomous Region	2021.3 Tibet Autonomous Region	2022.3 Xinjiang Uygur Autonomous Region	−	−	−	−	−	−
97	98	99	trachea	trachea	trachea	2025.2 Xinjiang Uygur Autonomous Region	2025.2 Xinjiang Uygur Autonomous Region	2025.2 Xinjiang Uygur Autonomous Region	−	−	−	−	−	−
100			swab			2025.2 Xinjiang Uygur Autonomous Region			+			28.63		

## Data Availability

The original contributions presented in the study are included in the article; further inquiries can be directed to the corresponding author.
